# Relapse prediction in schizophrenia with smartphone digital phenotyping during COVID-19: a prospective, three-site, two-country, longitudinal study

**DOI:** 10.1038/s41537-023-00332-5

**Published:** 2023-01-27

**Authors:** Asher Cohen, John A. Naslund, Sarah Chang, Srilakshmi Nagendra, Anant Bhan, Abhijit Rozatkar, Jagadisha Thirthalli, Ameya Bondre, Deepak Tugnawat, Preethi V. Reddy, Siddharth Dutt, Soumya Choudhary, Prabhat Kumar Chand, Vikram Patel, Matcheri Keshavan, Devayani Joshi, Urvakhsh Meherwan Mehta, John Torous

**Affiliations:** 1 Division of Digital Psychiatry, Beth Israel Deaconess Medical Center, Harvard Medical School, Boston, MA USA; 2 Department of Global Health and Social Medicine, Harvard Medical School, Boston, MA USA; 3 Department of Psychiatry, National Institute of Mental Health and Neurosciences (NIMHANS), Bengaluru, Karnataka India; 4 Sangath, Bhopal, India; 5 Department of Psychiatry, AIIMS Bhopal, All India Institute of Medical Sciences Bhopal, Bhopal, India

**Keywords:** Biomarkers, Diseases

## Abstract

Smartphone technology provides us with a more convenient and less intrusive method of detecting changes in behavior and symptoms that typically precede schizophrenia relapse. To take advantage of the aforementioned, this study examines the feasibility of predicting schizophrenia relapse by identifying statistically significant anomalies in patient data gathered through mindLAMP, an open-source smartphone app. Participants, recruited in Boston, MA in the United States, and Bangalore and Bhopal in India, were invited to use mindLAMP for up to a year. The passive data (geolocation, accelerometer, and screen state), active data (surveys), and data quality metrics collected by the app were then retroactively fed into a relapse prediction model that utilizes anomaly detection. Overall, anomalies were 2.12 times more frequent in the month preceding a relapse and 2.78 times more frequent in the month preceding and following a relapse compared to intervals without relapses. The anomaly detection model incorporating passive data proved a better predictor of relapse than a naive model utilizing only survey data. These results demonstrate that relapse prediction models utilizing patient data gathered by a smartphone app can warn the clinician and patient of a potential schizophrenia relapse.

## Introduction

Predicting and preventing relapse in psychosis remains a clinical priority, especially for patients with early course illness^1^. Relapse hampers recovery from schizophrenia, increases admissions to hospitals, contributes to the development of treatment-resistant schizophrenia, increases the risk of self-harm and homelessness, and impacts educational, vocational, and social functioning. The emotional and financial burden of relapse on caregivers and families of the patient has also been well documented^2,3^. Yet while relapse is common, impacting up to 20% of patients living with schizophrenia per year, it remains challenging to predict given the unique and dynamic social, personal, and environmental triggers that impact each patient differently^4^.

Digital technology, including social media^5^ and smartphones, offers a solution for predicting relapse given the ability to capture longitudinal, multimodal, and temporal dense relevant data to personal triggers for relapse^4^. Often referred to as digital phenotyping, this method involves using sensors in the smartphone that patients already typically own and use on a daily basis to capture information on their environment (e.g., green space exposure derived from GPS), social surroundings (e.g., degrees of contact based on call/text logs), personal behaviors (e.g., sleep duration based on accelerometer and screen time) and more^6^. Smartphones are also capable of capturing longitudinal assessments of symptoms^7^ and medication adherence through surveys, both of which can help us quantify the risk of relapse^8,9^. However, the automatic nature of capturing sensors has the distinct advantage of being independent from the user’s active engagement (such as taking surveys) and thus presents a more feasible means to passively capture the longitudinal data necessary to detect relapse^4^.

**Fig. 1:**
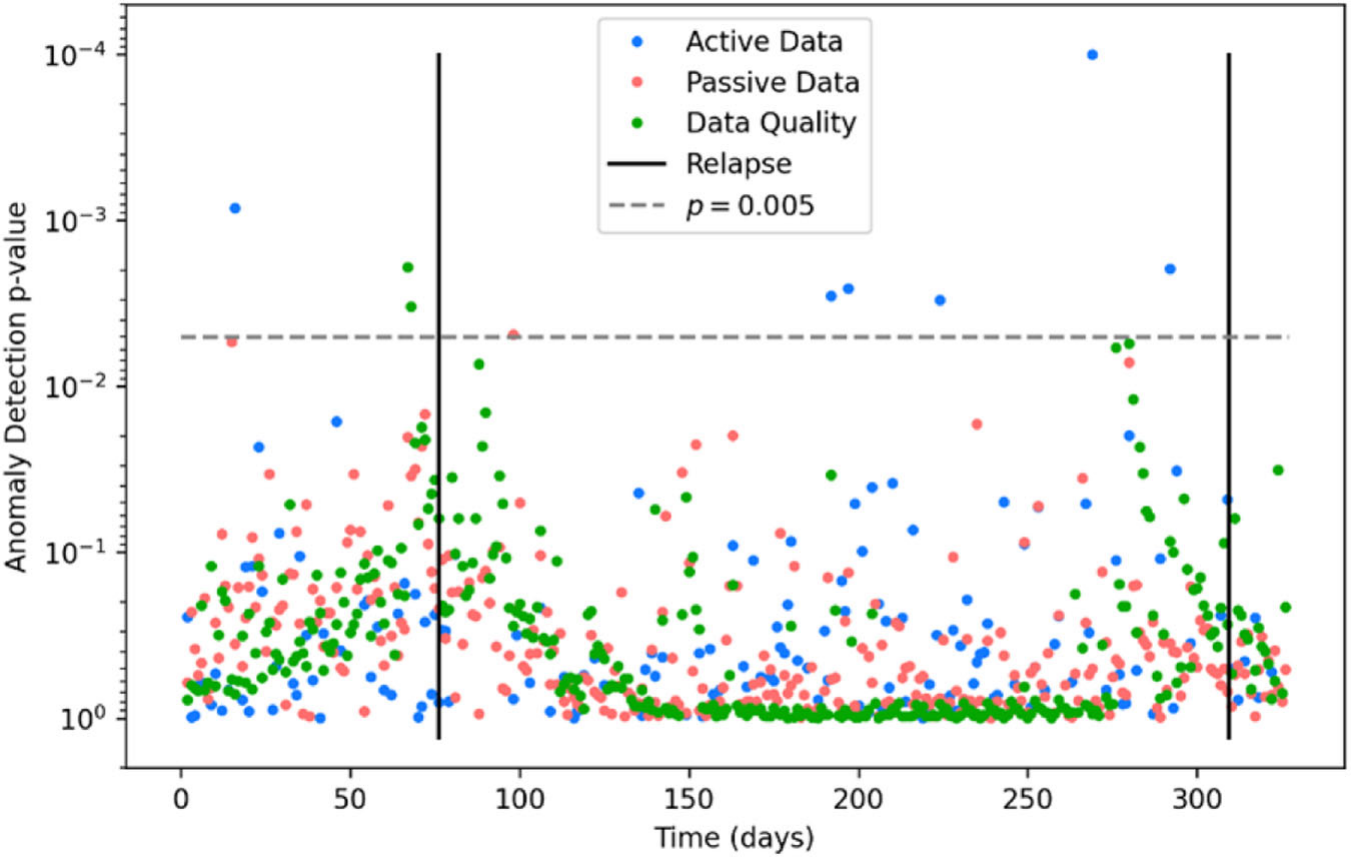
Sample individual participant anomaly detection plot. The *x* axis depicts the time in days since the first data point, while the *y* axis depicts the anomaly detection *p* value plotted inversely logarithmically. Solid black lines represent relapse events. The dotted gray line represents the *p* = 0.005 cutoff we chose for anomalies. Blue points represent active data, red points represent passive data, and green points represent data quality.

**Fig. 2:**
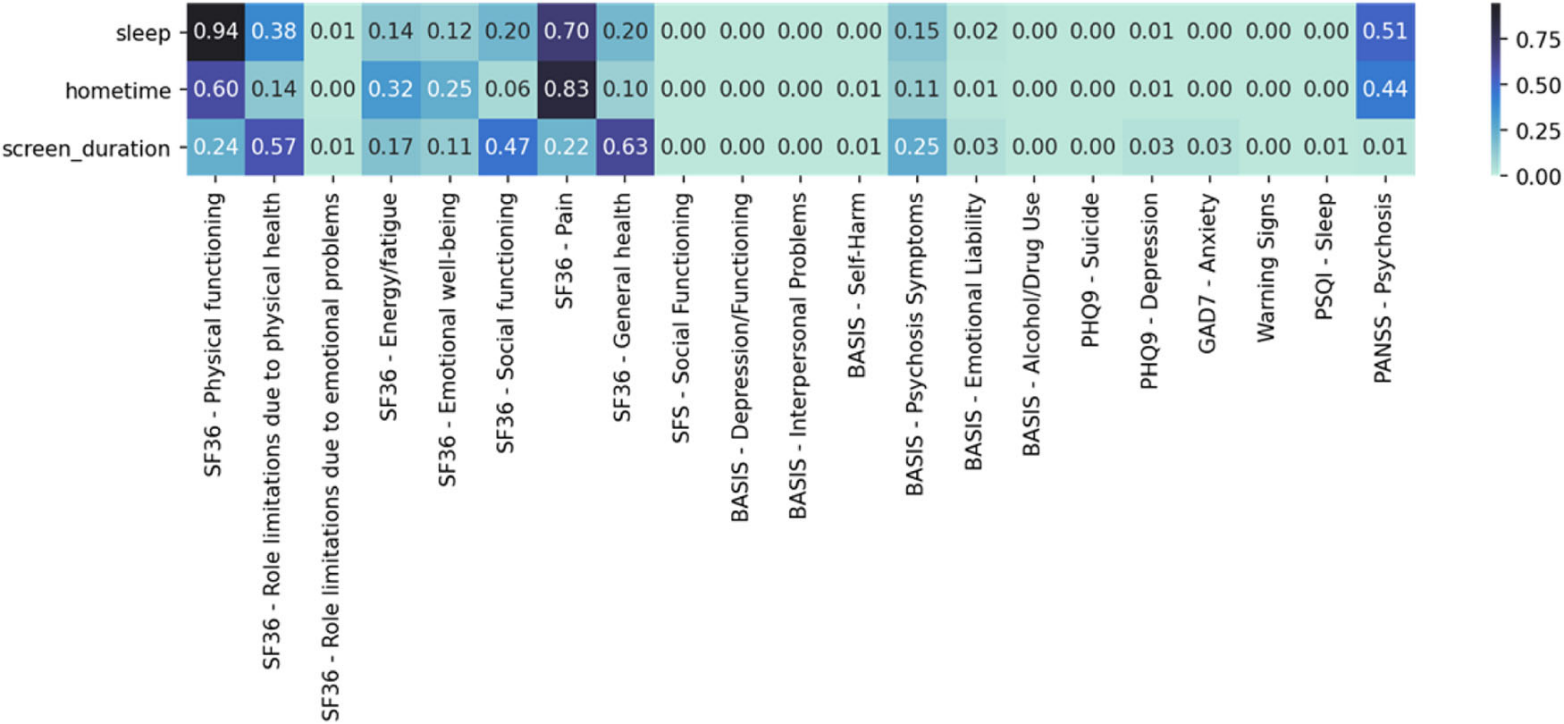
Correlation *p* values between passive data changepoints and symptom changes. Low *p* values indicate a strong correlation. A large amount of statistically significant *p* values illustrates the high association between passive data changepoints and changes in symptomatology.

Using both sensor and survey data for relapse prediction in psychosis is highly feasible. Our team has conducted two pilot studies and previously reported that using this digital phenotyping data with an anomaly detection algorithm can offer results with high sensitivity and specificity^10,11^. These algorithms allow researchers and clinicians to identify abnormal deviations from an individual’s typical sensor and survey data, which has been shown to be indicative of relapse. More than ten studies proposing relapse prediction metrics and algorithms based on the older CrossCheck dataset^12^ have suggested alternative innovative means to use digital phenotyping data for relapse prediction in schizophrenia. Recent studies have also successfully incorporated smartphone surveys into relapse prediction response systems^13^, suggesting clinical potential for combining surveys with data passively and automatically captured by digital phenotyping Figs. 1, 2.

However, for digital phenotyping methods to reach their potential in relapse prediction, they need to be scalable and work across diverse populations, contexts, and cultures^14^. By their very nature, smartphone-based healthcare tools should be readily sharable and broadly deployable. To date, no study has examined any digital phenotyping apps for relapse prediction in schizophrenia across different cultures and populations to assess if these systems can scale beyond use by the teams that developed them and provide results similar to those from promising pilot studies. Thus, we undertook this study to assess how a digital phenotyping relapse prediction system performed when used across three diverse sites: a rural site in India, an urban site in India, and an urban site in the United States.

Given the limited access to care for people living with schizophrenia in India, digital phenotyping relapse prediction offers important benefits. Prevention efforts are fundamental to the World Health Organization’s Mental Health Gap Action Program (mhGAP)^15^ and for such efforts to be effective they would need reliable and scalable metrics to guide the deployment of limited healthcare resources. While not the focus of this paper, accurate digital phenotyping of relapse via smartphone is appealing as it can support closed-loop interventions where the same smartphone can then respond to the risk by offering the user real-time and relevant app-based support^16^. But the foundation of such a system requires innovation in mental health monitoring and evaluation, which is the focus of this project.

The Smartphone Health Assessment for Relapse Prevention (SHARP) study aimed to explore the feasibility of digital phenotyping for relapse prediction across different regions, cultures, and languages. The protocol for the project has been published previously^17^, and the app was co-designed with patients, family members, and clinicians across all three sites^14^. The study was scheduled to begin on April 1^st^ 2020, but with changes in research regulation around the start of the COVID-19 pandemic and challenges with coordinating international efforts, the study was delayed. Given the pilot nature of this study to assess the performance of digital phenotyping for relapse prediction across diverse sites and concerns that the triggers and factors related to relapse are rapidly changing as COVID-19 restrictions are lifted in mid-2022, we chose to report on this first half of the ongoing data collection. The goal of this study was to explore the feasibility of anomaly detection in predicting relapse in patients with psychosis across three different sites. Building on recent promising evidence using these methods, we hypothesized that this approach would not only be feasible but also yield consistent findings at each site. Additionally, in this study, we explored whether passive digital data could predict symptom change among participants with psychosis.

## Results

### Demographics

Of the total participants (*N* = 132), 76 participants had a schizophrenia diagnosis (SZ) while 56 were healthy controls (HC). All 132 active and control participants across the three sites were enrolled in the study for a mean of 156 days with a standard deviation of 65 days. The participants in Boston were enrolled in the study for a mean of 145 days with a standard deviation of 80 days. The participants at Bangalore were enrolled in the study for a mean of 195 days with a standard deviation of 66 days while the Bhopal group of participants were enrolled in the study for a mean of 126 days with a standard deviation of 18 days. Participant demographic characteristics are detailed in Table 1.

**Table 1:** Characteristics of the participants.

Sample characteristics (*n*)	All sites (132)	Boston (33)	Bangalore (49)	Bhopal (50)	*p* value
Age (years), mean (SD)	32.33 (8.12)	37.84 (10.41)	30.82 (5.81)	35.32 (14.72)	0.001^a^
Missing	1	1	0	0	
Sex					0.004^b^
Female	58 (43.9%)	21 (63.6%)	17 (34.7%)	20 (40%)	
Male	72 (54.5%)	10 (30.3%)	32 (65.3%)	30 (60%)	
Other	2 (1.5%)	2 (6.1%)	0 (0%)	0 (0%)	
Missing	0 (0%)	0 (0%)	0 (0%)	0 (0%)	
Race					<0.001^b^
African American	6 (4.5%)	6 (18.2%)	0 (0%)	0 (0%)	
Asian	98 (74.2%)	0 (0%)	49 (100%)	49 (98%)	
Multiracial or other	5 (3.8%)	5 (15.2%)	0 (0%)	0 (0%)	
White	22 (1.7%)	21 (63.6%)	0 (0%)	1 (2%)	
Missing	1 (0.8%)	1 (3%)	0 (0%)	0 (0%)	
Ethnicity					<0.001^b^
Not Hispanic or Latino/a	123 (92.5%)	24 (70.6%)	49 (100%)	50 (100%)	
Hispanic or Latino/a	8 (6.1%)	8 (23.5%)	0 (0%)	0 (0%)	
Missing	1 (0.8%)	1 (2.9%)	0 (0%)	0 (0%)	
Education					<0.001^b^
Eighth Grade or Less	6 (4.5%)	0 (0%)	0 (0%)	6 (12%)	
Some High School	8 (6.1%)	0 (0%)	4 (8.2%)	4 (8%)	
High School Graduate/GED	20 (15.2%)	3 (9.1%)	12 (24.5%)	5 (10%)	
Some college	48 (36.4%)	18 (54.5%)	4 (8.2%)	26 (52%)	
4-year college graduate or higher	49 (37.1%)	11 (33.3%)	29 (59.2%)	9 (18%)	
Missing	1 (0.8%)	1 (3%)	0 (0%)	0 (0%)	

^a^Kruskal–Wallis rank sum test; ^b^Freeman–Halton test.

### Relapse Overview and Data Quality

In total, 20 participants experienced clinical relapses prior to August 1st 2022. 17 participants had one relapse, while three participants had two. Five relapses were related to reports of hospitalization, 2 were in connection with suicidal attempts or significant suicidal ideations, 4 were detected via 25% increases in monthly Positive and Negative Syndrome Scale (PANSS) scores, and the remainder, 9, were related to sudden and significant increases in psychosis symptoms requiring clinical intervention (e.g., medication dose increase). All relapses were either identified through self-report during monthly in-person or virtual consultations or by accessing medical records each month. The distribution of relapses across all three sites is depicted below (see Table 2). Using the Python package scipy, a chi-squared test was performed on the number of patients who experienced a relapse and the number of patients who did not relapse during the study across the three sites. The results suggest there was no statistically significant variation in the frequency of relapses across the three different sites (*χ*^2^ = 3.98, *df* = 2, *p* = 0.14).

**Table 2:** Number of participants in each site and in each participant group.

	Boston	Bangalore	Bhopal	Total
R	9	8	3	20
NR	17	17	22	56
C	7	24	25	56
Total	33	49	50	132

R represents the group of participants with schizophrenia who relapsed. NR is the group of participants with schizophrenia who did not relapse. C is the group of healthy controls.

In this study, data quality was defined as the ratio between the number of actual data points collected and the number of data points that would have been collected if all participants had completed all required surveys and all participant smartphones had transmitted all relevant passive data. The data quality across all three SHARP sites was relatively uniform, with an average active data quality of 28.5% and average passive data quality of 57.4%, see Table 3.

**Table 3:** Data quality analysis across three sites and three participant groups.

Active Data Quality					Passive Data Quality				
	Boston	Bangalore	Bhopal	Total		Boston	Bangalore	Bhopal	Total
R	0.321	0.360	0.118	0.326	R	0.555	0.611	0.660	0.591
NR	0.277	0.238	0.311	0.271	NR	0.533	0.519	0.623	0.555
C	0.303	0.225	0.339	0.280	C	0.840	0.482	0.642	0.585
Total	0.298	0.258	0.316	0.285	Total	0.596	0.523	0.635	0.574

R represents the group of participants with schizophrenia who relapsed. NR is the group of participants with schizophrenia who did not relapse. C is the group of healthy controls.

### Anomaly Detection

Multivariate anomaly detection software was run on each user’s data individually to quantitatively determine the likelihood that each measurement was atypical compared to temporally nearby data points. The data streams were those collected actively, or passively, and those which represented data quality. From these calculations, 132 anomaly detection plots were generated, one for each participant.

Anomaly detection analyses revealed 188 significant anomalies at *p* = 0.005. A lower *p* value was chosen to accommodate the rarity of relapse events. 13 anomalies (6.9%) were true positives Table 4.

This anomaly detection model was compared to a more naive logistic regression trained on demographic data, participant-reported medication adherence data, and participant-reported psychosis symptoms data. Both models were run on the data from each site individually as well as all sites simultaneously to compare their efficacy at predicting relapse events.

**Table 4:** Summary statistics for both naive logistic regression model (left) and anomaly detection model (right).

Naive logistic regression statistics					Anomaly detection statistics				
	BIDMC	Bangalore	Bhopal	Total		BIDMC	Bangalore	Bhopal	Total
RMSE	0.304	0.224	0.284	0.271	RMSE	0.268	0.180	0.125	0.191
Sensitivity	0.000	0.200	0.000	0.048	Sensitivity	0.009	0.004	0.004	0.006
Specificity	0.990	0.967	0.933	0.968	Specificity	0.996	0.997	0.999	0.997
TP	0	1	0	1	TP	9	3	1	13
FP	1	7	9	14	FP	54	97	24	175
TN	98	208	125	427	TN	13314	27698	18540	59552
FN	9	4	2	20	FN	978	834	269	2081

RMSE, TP, FP, TN, and FN stand for root mean squared error, true positive, false positive, true negative, and false negative respectively.

Overall, anomalies were 2.12 times more frequent in the month preceding and 2.78 times more frequent in the month preceding and following a relapse, as compared to other timeframes. By contrast, the logistic regression model predicted a relapse event 1.5 times as frequently in months containing a relapse event compared to other months. In other words, when predicting schizophrenia relapse within a 30-day timeframe, the anomaly detection model was 1.41 times more effective than the logistic regression model.

Additionally, permutation testing was utilized to evaluate the plausibility of the null hypothesis, the suggestion that anomaly detection was equally effective at all three sites. We evaluated whether the receiver operator characteristic (ROC) curves observed amongst the control participants in our study were significantly different from those obtained if the site labels of each control participant were randomly permutated. This resulted in a *p*-value of 0.165.

### Changepoint detection

Statistical analyses were conducted to determine if changepoints in passive data streams were correlated with symptom change as measured by monthly clinical scales quizzes. Significant correlations were found between passive data changepoints and a variety of scores related to psychosis symptoms as measured with the Positive and Negative Syndrome Scale (PANSS) for Schizophrenia^18^, depression with the Patient Health Questionnaire-9 (PHQ-9)^19^, anxiety with the Generalized Anxiety Disorder-7 (GAD-7)^20^, quality of life with the short-form health scale (SF36)^21^ social functioning with (SFS)^22^, sleep quality of the Pittsburgh Sleep Quality Index (PSQ)^23^, warning signs of relapse with the Warning Signals Scale (WSS)^24^, questions about symptoms including substance abuse from the Behaviour and Symptom Identification Scale–Revised (BASIS-24)^25^.

## Discussion

This study explored anomaly detection as a statistical method to predict relapses in patients with psychosis symptoms in three different sites: an urban city in the United States, an urban city in India, and a more rural setting India. Overall, across all three SHARP sites, there was a significant association between the number of statistically significant data anomalies and psychosis relapses.

These results support anomaly detection as a viable method for predicting schizophrenia relapse. Given the heightened correlation between anomalies and relapses both prior to and following relapses, we conclude that anomaly detection not only picks up on the relapse itself, but also allows us to track entire psychotic episodes in a data-driven way. This not only corroborates but also complements prior literature^10,11^ both by reflecting the reality that individuals may stay ill for extended periods of time after their initial relapse, but also accounting for several new variables, most notably cultural and language differences. Furthermore, our permutation testing analysis failed to reject the null hypothesis that anomaly detection was equally effective at predicting relapse at all three sites. In other words, the percentage increases in anomalies detected around a relapse at all three sites were statistically identical at a significance level of *α* = 0.05. The above results suggest that anomaly detection and the digital phenotyping data fueling it may be able to capture universal features of psychotic illnesses that are invariant across global sites and cultures. However, it does not mean all people with the illness and sites are the same, and the *p* value of 0.165 indicates there were likely minor differences in the algorithm’s effectiveness between the three sites. A plausible explanation is that COVID-19 affected the three sites very differently, in terms of extended lockdowns and restrictions on in-person activities, leading to slightly different digital phenotyping patterns. Cultural differences and environmental factors not captured by the smartphone also offer another contributor.

Although active data—most notably psychosis surveys and medication adherence surveys—historically provides good predictive tools for anticipating schizophrenia relapses^4^ this study shows that complementing these active data streams with passive data and data quality metrics improved predictive power by a quantitatively measurable degree of 1.41 times.

Various factors affected our data quality. For active data quality, while participants received reminders from mindLAMP to complete surveys, this was no guarantee that the surveys would be completed. Several participants may have simply ignored the reminders while others may have seen the reminders but forgot to fill out the surveys. Lastly, participants may have turned off app notifications. Some plausible explanations for the passive data quality include participants forgetting to charge their phone and turning on low power mode, both of which disrupts the mindLAMP’s data collection. Additionally, participants, although instructed not to, may have turned off their GPS. For these reasons it is often difficult to achieve perfect passive data quality. Despite all these factors, our passive data quality was comparable to or even exceeded that of several other studies^26–28^.

Another advantage of anomaly detection is that it identifies which passive data streams were most responsible for producing the anomalous reading. This gives anomaly detection extra clinical significance: as soon as the algorithm identifies an anomalous event, the clinical team could aim to prevent relapse by offering immediate care or digitally providing early therapeutic intervention tailored specifically to each of the passive data streams that was found to be anomalous. The fact that we did not observe a specific data stream (e.g., sleep) that was most predictive of relapse across all individuals is likely indicative of the widely varying symptomatologies exhibited by individuals with schizophrenia and the personalized nature of relapse (as well as recovery).

Finally, through participants’ clinical scales data, we demonstrate that changepoints in passive data are correlated with self-reported symptom change. In other words, changepoint detection provides encouraging results in predicting variations in clinical data within a 30-day period. Changepoint detection may prove practical in care because smaller changes in symptoms and behaviors are more common than relapse events which are typically rare. However, additional research specifically designed to investigate the efficacy of changepoint detection is necessary to confirm these preliminary findings.

It is challenging to compare our results to prior studies. Data from CrossCheck has been used in many research papers but represents information that is older and no longer feasible to obtain due to changes around data availability rules from smartphone manufacturers. But as a historical comparison, we note our results are in line with more recent results^11^, which found a 71% increase in the rate of anomalies two weeks around relapse, as compared to the 112% increase a month prior to relapse reported in the current study. No other study to this date has made a comparison between an anomaly detection model and an algorithm incorporating only active data.

This study has several key limitations. All relapse data was obtained from clinical interviews and medical records, but it is possible some participants may have had relapses between interviews and not reported them when asked. Another limitation of this study is COVID-19 and our data reflecting how those participants in the study felt and behaved in the setting of restrictions on mobility and usual life. As such, smartphone behavior and overall data quality may not reflect regular usage outside of this time frame, affecting generalizability of our passive data results. Additionally, the impact of and response to COVID-19 varied between the United States and India as do cultural and environmental factors between all sites. This may have impacted the sites and the participants at each site differently. For example, lockdown periods may have varied by duration and the exact time it took place. Even within the United States, the degree of lock down and social distancing varied depending on the county and state. As a result, participants’ passive data, a reflection of behavioral patterns, were most likely impacted differently across the three sites. However, once this study concludes, the data collected early during COVID-19 pandemic will be compared to the data collected later in the pandemic (notably after restrictions on in-person activities were lifted) to note any differences in the efficacy of the anomaly detection algorithm.

## Methods

### Recruitment

Participants were recruited at three different sites: Beth Israel Deaconess Medical Center (BIDMC) in Boston, USA, the National Institute of Mental Health and Neuroscience (NIMHANS) in Bangalore, India, and the Sangath Bhopal Hub jointly with the All India Institute for Medical Sciences (AIIMS) in Bhopal, India. Participants in Bangalore were recruited through outpatient services within the NIMHANS hospital while participants in Bhopal were recruited by the AIIMS outpatient psychiatry.

To participate in the study, participants were required to be diagnosed with a psychotic spectrum disorder or to have experienced psychosis within 5 years before September 2021, when the study began. This was confirmed by a clinician using DSM-5 criteria. All participants were required to be in active treatment and have access to a smartphone (Android or iPhone) with cellular service or wifi. The smartphone needed to be compatible with mindLAMP and continuously collect adequate data throughout the whole study as determined by the research team. If a patient at either of the India sites did not have a compatible smartphone, they were provided with a Samsung Galaxy M31 that had cellular service. To ensure that the participant’s phone was collecting data, participants had to go through a one-week trial period in which data collection was measured. If participants had multiple days of no passive data collection, they did not pass the trial period. Controls were age, sex, and education matched to the experimental participants. All sites received ethics approval from respective their Institutional Review Boards (IRBs): Beth Israel Deaconess Medical Center, Sangath IRB and All India Institute of Medical Sciences Bhopal Institutional Human Ethics Committee (IHEC), and the National Institute for Mental Health and Neurosciences IHEC. All participants provided written informed consent.

### Protocol

The protocol for this study has been published^17^. Each month, participants had an hour-long in-person visit at NIMHANS and Sangath-AIIMS or virtual visit at BIDMC with a research assistant. The virtual visits were conducted over StarLeaf, a HIPAA compliant video conferencing platform.

During each visit, a research assistant administered the PANSS, a measure of psychosis symptoms^18^. Participants were then instructed to complete the following surveys within 24 hours of each visit: Patient Health Questionnaire-9 (PHQ-9)^19^, the Generalized Anxiety Disorder-7 (GAD-7) survey^20^, Short Form (SF-36) survey^21^, the Social Functioning Scale (SFS)^22^, the Pittsburgh Sleep Quality Index (PSQI)^23^, Warning Signal Scale (WSS)^24^, Behavior and Symptom Identification Scale (BASIS-24)^25^. The Brief Assessment of Cognition in Schizophrenia (BACS)^29^ survey was only administered at the intake visit, 6-month visit, and 12-month visit. All of the above surveys were administered through REDCap surveys at BIDMC or by paper and pen at Sangath-AIIMS and NIMHANS. Inter-rater reliability for the PANSS was examined for research assistants administering the PANSS from each of the three sites by having them rate five video-recorded clinical interviews—once at the start of the study and once again at 6 months. Intraclass correlations were excellent (>0.75) for PANSS Total and Positive scores and fair to good (>0.4) for PANSS Negative scores^30^.

Participants were provided with monetary compensation. For the 1-year study at the Boston site, they were paid $50 at visits 1, 7, and 13 and $20 at all other visits. This totaled up to $350. For the 3-month study, participants received $30 for each visit, totaling $120 for all four visits. At the Indian sites, participants were paid between 500 to 2000 rupees for each visit with compensation depending on the distance traveled. However, across all sites, no compensation was provided for app engagement itself or for the collection of active and passive data.

### MindLAMP

Participants were instructed to use mindLAMP, an open-source smartphone app developed by the Division of Digital Psychiatry at Beth Israel Deaconess Medical Center^31^ in collaboration with patients with schizophrenia, their family members, and clinicians at each of the three sites. MindLAMP collected both the active data and passive data for participants.

The active data consisted of six surveys: PHQ-9, GAD-7, sleep, sociability, psychosis, and medication adherence. The sleep, sociability, and psychosis surveys were modified from the PSQI, Social Functioning Scale, and PANSS respectively. MindLAMP sent participants notifications for two of the above six surveys, selected randomly, twice a day. This was done in hopes that the participant would complete the four random surveys each day at the time the notification was sent, thus reducing recall bias. Active data quality was calculated by dividing the number of surveys completed per participant by the expected number of surveys completed each day.

The three passive data streams were accelerometer, GPS, and screen state. The app also provided psychoeducational content, audio-guided meditations, journaling, and other activities. Research assistants provided any app-related technical support and encouraged participants to complete activities through mindLAMP.

Cortex^32^, the open-source and built-in data analysis toolkit for mindLAMP, was used to extract data from the smartphone app and manipulate it to a format, which was compatible with the anomaly detection code. It was also used to calculate derived metrics such as data quality, sleep duration, time at home, and screen duration from raw passive data streams. All of the above was done in Python except for the anomaly detection calculations, which were performed in R and C++.

### Clinical targets

In accordance with prior research, relapses were pre-defined as one of the following: (1) a 25% increase in a participant’s PANSS score, (2) a psychiatric hospitalization, (3) a suicidal attempt or a significant and sudden increase in suicidal ideation, (4) a significant or sudden increase in psychosis symptoms requiring clinical intervention. All the above criteria were assessed via monthly in-person or virtual consultations or by accessing medical records.

### Anomaly detection

Multivariate anomaly detection was used to longitudinally analyze all data streams simultaneously to detect signs of relapse. All data streams were aggregated to a daily time scale. Then, given the overall data, each day was associated with a *p* value quantifying how anomalous that day’s data points were. For a detailed mathematical description of how these *p* values were calculated, please refer to prior literature^11^ and the publicly available code written in R and C++ (https://www.notion.so/digitalpsychiatry/Anomaly-Detection-in-R-177a40b5120343fdad1bff6db7632118).

This study classified events with a *p*-value less than or equal to 0.005 as formal anomalies. This struck a balance between the canonical 0.05 cutoff and a traditional Bonferroni correction, which would classify all events as non-anomalies given the large number of tests performed.

An anomaly was considered a true positive if it occurred within 30 days of a relapse event. The data streams were first grouped by the method of collection—namely active, passive, or data quality. They were subsequently grouped by the clinical significance of each data stream. These different feature groups were (1) symptoms, (2) sociability, (3) medication, (4) sleep, (5) hometime & screen duration, and (6) engagement.

This anomaly detection model was compared to a naive logistic regression model trained on purely active data. For each month and each participant, the logistic regression model was trained to use a participant’s demographic data, medication adherence survey scores, and psychosis symptom scores, to detect whether a relapse had occurred in that month. Several summary statistics such as root mean squared error, sensitivity, and specificity were calculated for both models. Finally, both models were judged by how much more frequently they predicted a positive result in the 30 days surrounding a relapse as opposed to other timeframes during the study.

After applying the anomaly detection model to each site individually, permutation testing was utilized to discern if the increase in the number of anomalies detected were statistically similar at the three sites. For each site, we calculated the area underneath an ROC curve obtained by plotting the false positive rate versus different anomaly detection cutoff *p* values for the control participants. False positive rates above 25% were ignored because we judged that false positive rate above 25% are not clinically tolerable. Then under the null hypothesis, we expected there to be very little difference in the spread of these ROC statistics if the site label (i.e., BIDMC, Bangalore, Bhopal) for each control participant was randomly permuted. Permuting the control participants allowed us to test both the exchangeability of the site label as well as how equal the effect magnitude is across the three sites. After randomly sampling 1000 permutations, the proportion of permutations with a more extreme spread than the observed spread was the chosen *p*-value.

### Changepoint detection

Changepoint detection was used as supplementary experimental analysis to explore if passive data could be used as a predictor of symptom change. Independently, the package sdt-python was used to analyze each participant’s passive data by implementing the PELT changepoint detection algorithm with an L2 cost function^33^. For each passive data stream and each participant, this algorithm gave a list of days on which the relevant participant exhibited statistical changepoints in the relevant passive data stream.

Finally, using the Python pandas function pandas.DataFrame.corr, Pearson correlation coefficients were calculated to determine if survey scores were correlated to the presence of a passive data changepoint within 10 days of the relevant questionnaire. The scipy.stats.pearsonr method was used to calculate the associated *p* values.

## Conclusion

This study supports the feasibility of utilizing smartphone-based anomaly detection to predict schizophrenia relapse in patients across multiple study sites in the United States and India. Furthermore, our results illustrate that using both passive and active data streams provides for more robust prediction models. However, future research must be conducted to elucidate how anomaly detection models can be integrated effectively into clinical care.

## Data Availability

The data are not publicly available due to the nature of the IRB approval and participant privacy concerns around geolocation data. Please contact the corresponding author for access.

## Code availability

The code for anomaly detection is publicly available at https://www.notion.so/digitalpsychiatry/Anomaly-Detection-in-R-177a40b5120343fdad1bff6db7632118.

## References

[1] Jauhar, S., Laws, K., Fusar-Poli, P. & McKenna, P. Relapse prevention in schizophrenia. *Lancet Psychiatry* **9**, E13 (2022). 10.1016/S2215-0366(21)00501-0 35305747

[2] Kane JM (2007). Treatment strategies to prevent relapse and encourage remission. J. Clin. Psychiatry.

[3] Almond S, Knapp M, Francois C, Toumi M, Brugha T (2004). Relapse in schizophrenia: costs, clinical outcomes and quality of life. Br. J. Psychiatry.

[4] Henson P., Wisniewski, H., Stromeyer IV, C., & Torous J. Digital health around clinical high risk and first-episode psychosis. *Curr. Psychiatry Rep.* **22**, 58 (2020). 10.1007/s11920-020-01184-x 32880764

[5] Birnbaum, M. L. et al. Detecting relapse in youth with psychotic disorders utilizing patient-generated and patient-contributed digital data from Facebook. *Npj Schizophr*. **5**, 17 (2019). 10.1038/s41537-019-0085-9 PMC6779748 31591400

[6] Torous, J., Kiang, M. V., Lorme, J. & Onnela, J.-P. New tools for new research in psychiatry: a scalable and customizable platform to empower data driven smartphone research. *JMIR Ment. Health* **3**, e16 (2016). 10.2196/mental.5165 PMC4873624 27150677

[7] Liu G, Henson P, Keshavan M, Pekka-Onnela J, Torous J (2019). Assessing the potential of longitudinal smartphone based cognitive assessment in schizophrenia: a naturalistic pilot study. Schizophr. Res. Cogn..

[8] Jørgensen P (1998). Early signs of psychotic relapse in Schizophrenia. Br. J. Psychiatry.

[9] Robinson D (1999). Predictors of relapse following response from a first episode of schizophrenia or schizoaffective disorder. Arch. Gen. Psychiatry.

[10] Henson, P., D’Mello, R., Vaidyam, A., Keshavan, M. & Torous, J. Anomaly detection to predict relapse risk in schizophrenia. *Transl. Psychiatry* **11**, 28 (2021). 10.1038/s41398-020-01123-7 PMC7798381 33431818

[11] Barnett I (2018). Relapse prediction in schizophrenia through digital phenotyping: a pilot study. Neuropsychopharmacology.

[12] Wang, R. et al. CrossCheck: toward passive sensing and detection of mental health changes in people with schizophrenia. *UbiComp '16: Proceedings of the 2016 ACM International Joint Conference on Pervasive and Ubiquitous Computing**.*10.1145/2971648.2971740 (2016).

[13] Gumley AI (2022). The EMPOWER blended digital intervention for relapse prevention in schizophrenia: a feasibility cluster randomised controlled trial in Scotland and Australia. Lancet Psychiatry.

[14] Rodriguez-Villa E. et al. Cross cultural and global uses of a digital mental health app: results of focus groups with clinicians, patients and family members in India and the United States. *Glob. Ment. Health* **8**, e30 (2021). 10.1017/gmh.2021.28 PMC8392688 34512999

[15] World Health Organization. mhGAP intervention guide for mental, neurological and substance use disorders in non-specialized health settings: Mental Health Gap Action Programme (mhGAP) (2016). 23741783

[16] Wang L, Miller LC (2019). Just-in-the-Moment Adaptive Interventions (JITAI): a meta-analytical review. Health Commun..

[17] Rodriguez-Villa, E. et al. Smartphone health assessment for relapse prevention (SHARP): a digital solution toward global mental health. *BJPsych Open* **7**, E29 (2021). 10.1192/bjo.2020.142 PMC8058838 33407986

[18] Kay SR, Fiszbein A, Opler LA (1987). The positive and negative syndrome scale (PANSS) for schizophrenia. Schizophr. Bull..

[19] Kroenke K, Spitzer RL, Williams JBW (2001). The PHQ-9: validity of a brief depression severity measure. J. Gen. Intern. Med..

[20] Spitzer, R. L., Kroenke, K., Williams, J. B. W. & Löwe, B. Generalized anxiety disorder 7. 10.1037/t02591-000 (2011).

[21] Ware JE, Sherbourne CD (1992). The MOS 36-item short-form health survey (SF-36). I. Conceptual framework and item selection. Med. Care.

[22] Birchwood M, Smith J, Cochrane R, Wetton S, Copestake S (1990). The Social Functioning Scale. The development and validation of a new scale of social adjustment for use in family intervention programmes with schizophrenic patients. Br. J. Psychiatry.

[23] Buysse DJ, Reynolds CF, Monk TH, Berman SR, Kupfer DJ (1989). The Pittsburgh Sleep Quality index: a new instrument for psychiatric practice and research. Psychiatry Res..

[24] Jørgensen P (1998). Schizophrenic delusions: the detection of warning signals. Schizophr. Res..

[25] Cameron IM (2007). Psychometric properties of the BASIS-24© (behaviour and symptom identification scale–revised) mental health outcome measure. Int. J. Psychiatry Clin. Pract..

[26] Straczkiewicz M (2022). Combining digital pill and smartphone data to quantify medication adherence in an observational psychiatric pilot study. Psychiatry Res..

[27] Matcham, F. et al. Remote assessment of disease and relapse in major depressive disorder (radar-MDD): recruitment, retention, and data availability in a longitudinal remote measurement study. *BMC Psychiatry* **22**, 136 (2022). 10.1186/s12888-022-03753-1 PMC8860359 35189842

[28] Nickels, S. et al. Toward a mobile platform for real-world digital measurement of depression: user-centered design, data quality, and behavioral and Clinical Modeling. *JMIR Ment. Health* **8**, e27589 (2021). 10.2196/27589 PMC8386379 34383685

[29] Keefe R (2004). The Brief Assessment of Cognition in Schizophrenia: reliability, sensitivity, and comparison with a standard neurocognitive battery. Schizophr. Res..

[30] Kølbæk P (2018). Inter-rater reliability of ratings on the six-item Positive and Negative Syndrome Scale (PANSS-6) obtained using the Simplified Negative and Positive Symptoms Interview (SNAPSI). Nord. J. Psychiatry.

[31] Torous J (2019). Creating a digital health smartphone app and digital phenotyping platform for mental health and diverse healthcare needs: an interdisciplinary and collaborative approach. J. Technol. Behav. Sci..

[32] What is cortex?: Lamp platform. Lamp Platform RSS Available at: https://docs.lamp.digital/data_science/cortex/what_is_cortex/. (Accessed: 23rd September 2022).

[33] Killick R, Fearnhead P, Eckley IA (2012). Optimal detection of changepoints with a linear computational cost. J. Am. Stat. Assoc..

